# Assessment of urate-lowering therapies on lipid metabolism and kidney function in non-dialysis chronic kidney disease patients: 12 months multicenter cohort study

**DOI:** 10.3389/fendo.2025.1592290

**Published:** 2025-09-09

**Authors:** Yousuf Abdulkarim Waheed, Huanhuan Yin, Jie Liu, Shifaa Almayahe, Maryam Bishdary, Karthick Kumaran Munisamy Selvam, Syed Muhammad Farrukh, Shulin Li, Disheng Wang, Xinglei Zhou, Dong Sun

**Affiliations:** 1 Department of Nephrology, Affiliated Hospital of Xuzhou Medical University, Xuzhou, China; 2 Clinical Research Center for Kidney Disease Xuzhou Medical University, Xuzhou, China; 3 Department of Nephrology, Fengxian People’s Hospital, Xuzhou, China; 4 Department of Nephrology, the Second Affiliated Hospital of Xuzhou Medical University, Xuzhou, China; 5 Medical College, University of Fallujah, AL Anbar, Iraq; 6 Central Michigan University College of Medicine, Mount Pleasant, MI, United States; 7 Medical College, Xuzhou Medical University, Xuzhou, China; 8 Department of Internal Medicine and Diagnostics, Xuzhou Medical University, Xuzhou, China

**Keywords:** serum uric acid, chronic kidney disease, urate-lowering therapy, hyperuricemia, dyslipidemia, cardiovascular risk

## Abstract

**Background and objectives:**

Urate lowering therapies (ULTs) are primarily used to manage hyperuricemia (HUA), which refers to an increase in serum uric acid (SUA) levels. SUA is an important marker for assessing kidney function in patients complicated with chronic kidney disease (CKD). Recent studies revealed a close relationship between SUA and lipid metabolism. We aim to investigate the impact of ULTs on kidney function and lipid profiles in CKD patients, and further explore the sex-specific ULTs effects on lipid profiles.

**Method:**

We conducted a multicenter, prospective observational cohort study, enrolled n=200 patients aged between 20 and 80 years old with stages 3/4 CKD. Patients were divided into two groups: the ULT group (n=94) who were receiving febuxostat or allopurinol, and the Non-ULT group (n=106) who were receiving their conventional CKD therapy, the study employed clinically indicated allocation. ULT initiation was based on physician judgment per guidelines persistent HUA with SUA ≥7 mg/dL in males and ≥6 mg/dL in females with CKD progression risk factors. Models adjusted for all collected confounders, renal function including estimated glomerular filtration rate (eGFR), serum creatinine (Scr), blood urea nitrogen (BUN), and SUA, and lipid profiles including high-density lipoprotein cholesterol (HDL-c), low-density lipoprotein cholesterol (LDL-c), triglyceride (TG), and total cholesterol (TC). Results remained consistent in sensitivity analyses stratifying by baseline characteristics. Subgroups were further analyzed based on sex, to evaluate sex-specific differences in lipid metabolism related to ULTs. All participants went through clinical assessment before and after treatment and were followed for 12 consecutive months.

**Results:**

LDL-c significantly decreased in the ULT group compared to the Non-ULT group after 12 months of observation (2.14 ± 0.32 vs. 2.42 ± 0.32 [95% CI: -0.36 to -0.18], *P<0,001*). Similarly, TC and TG were significantly decreased in the ULT group compared to the Non-ULT group after 12 months of observation (4.18 ± 0.44 vs. 4.47 ± 0.39 [95% CI: -0.40 to -0.16], *P<0,001*) for TC, and (2.43 ± 0.62 vs. 2.63 ± 0.58 [95% CI: -0.37 to -0.03], *P<0,016*) for TG. Moreover, HDL-c increased significantly in the ULT group compared to the Non-ULT group (1.41 ± 0.13 vs. 1.23 ± 0.15 [95% CI: 0.13 to 0.21], *P<0.001*). The sex-specific ULT on lipid profiles exhibited a greater reduction in LDL-c in males by (-0.28 mmol/L [95% CI: -0.32 to -0.14], *P<0.001*), and a more pronounced increase in HDL-c levels by (+0.23 mmol/L [95% CI: 0.07 to 0.18], *P<0.001*). A significant correlation was observed Pre- and Post-treatment between SUA and LDL-c/HDL-c, Post-treatment LDL-c (R=0.2942, R²=0.2639, 95% CI: [0.0974 to 0.4689], *P<0.0040)*, Post-treatment HDL-c (R=-0.3935, R²=0.1548, 95% CI: [-0.5521 to -0.2074], *P<0.0001*). SUA significantly decreased in the ULT group compared to the Non-ULT group after 12 months of treatment (398.55 ± 45.48 vs. 456.66 ± 38.23 [95% CI: -69.78 to -46.42], *P<0.001).* Similarly, eGFR slightly improved in the ULT group compared to the Non-ULT after 12 months of treatment (40.83 ± 7.50 vs. 34.43 ± 7.68 [95% CI: 4.32 to 8.51], *P<0.001).* These results indicate the renoprotective effects of ULTs in CKD patients.

**Conclusion:**

In this cohort study of non-dialysis CKD patients, ULT use was associated with improved lipid profiles reduced LDL-c, TG, and TC; increased HDL-c, with greater HDL-c elevation and LDL-c reduction in males. ULTs exposure also correlated with attenuated CKD progression. These findings suggest potential interactions between SUA and lipid metabolism, highlighting ULTs’ possible role in managing dyslipidemia and renal function decline in pre-dialysis CKD.

## Graphical Abstract

**Figure:**
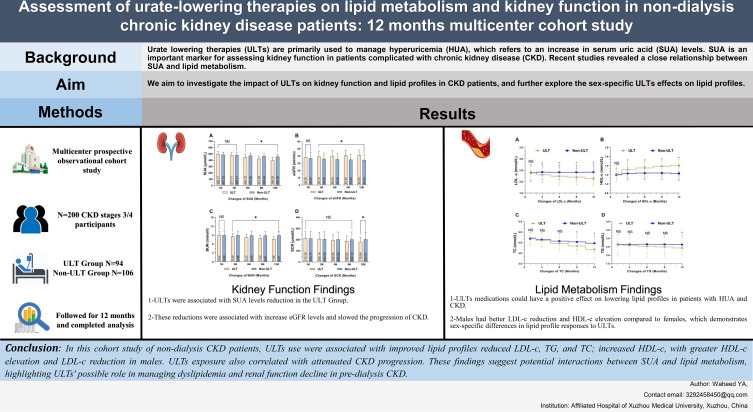

## 1 Introduction

Chronic kidney disease (CKD) is a significant public concern, not only in China but worldwide, affecting a large and growing proportion of the population. CKD is characterized by gradual loss of kidney function over time. CKD, if left untreated, not only will lead to end-stage kidney disease (ESKD) but also contribute to a heightened risk of developing cardiovascular disease (CVD) and overall mortality (1). Many factors can influence the progression of CKD, and these can be termed traditional factors, such as hypertension (HTN), and diabetes and nontraditional factors, such as inflammation, oxidative stress, and mineral bone disorders (2). As CKD is known to be a significant health burden with several dimensions, it is important to discover new treatment targets or strategies that would slow down the CKD progression and improve the overall prognosis. This explains why such high-persistent diseases should draw the attention of early diagnosis and intervention for effective management.

The end-product of purine metabolism is serum uric acid (SUA), produced in the liver and ultimately excreted via the kidneys throughout the body (3–5). Mostly, it is formed by endogenous synthesis with less being sourced externally (6). Abnormalities in either excessive production or under-excretion define the causes behind hyperuricemia (HUA). The definition for HUA diagnosis in China states the cut-off value for SUA concentration > 420 μmol/L, specifically applied to male and female patients (7). According to one meta-analysis, the estimated aggregated prevalence of HUA in mainland China was found to be 13.3% (95% CI: 11.9-16.4%) (8). SUA is a critical factor in CKD progression through multiple pathways: it has independent associations with the risk of decline in renal function, all-cause mortality, and cardiovascular events, especially in the later stages of CKD (5). Both high and very low SUA concentrations exhibit U-shaped relationships with mortality with inflection points of (311.65 μmol/L all-cause and 392.34 μmol/L CVD) (9). HUA, >420 μmol/L in China is associated with tubulointerstitial damage, faster estimated glomerular filtration rate (eGFR) decline, and coronary calcification in early CKD (10, 11). Mechanisms and metabolism of SUA and HUA have been previously published in our reviews in greater detail (4, 5).

Due to the inability of the kidneys to metabolize and eradicate lipids in CKD patients, this process will lead to the deposition of atherogenic lipoproteins; dyslipidemia in CKD is characterized by highly augmented serum low-density lipoprotein cholesterol (LDL-c) and oxidized LDL (OX-LDL) levels. The accumulated levels of LDL-c are indicative of dyslipidemia related to CKD. OX-LDL is simply a modified form of LDL-c, with great detrimental effects due to its inductive properties for inflammation and the formation of foam cells in renal blood vessels (12). The modified lipoprotein is taken up by the scavenger receptors on macrophages, with foam cell formation, culminating in glomerular damage (13). Hypertriglyceridemia is also a common occurrence in the CKD population, due simply to impaired lipoprotein lipase activity and increased triglyceride-rich lipoproteins harboring insulin resistance, which is often prevalent in CKD patients (14, 15). Furthermore, there is a low level of high-density lipoproteins cholesterol (HDL-c) in CKD patients, thereby overturning its protective effects against inflammation, oxidative damage, and cholesterol accumulation. HDL-cin CKD patients is dysfunctional, compared to those without kidney disease which arrests the reverse transport of cholesterol and ameliorates oxidative stress (16). In sum, these various lipid disorders provide services to set a stage for inflammatory and oxidative processes most conducive to renal injury and cause CKD to progress rapidly. The condition of dyslipidemia in CKD can lead to the activation of the renin-angiotensin-aldosterone system (RAAS), a major contributor to renal impairment. Angiotensin II, a dominant component of the RAAS, can promote vasoconstriction, sodium retention, and the release of pro-fibrotic elements such as transforming growth factor-beta (TGF-β) (17). These results can initiate glomerular sclerosis and tubulointerstitial fibrosis, which can significantly speed the progression of CKD (18). The dyslipidemia-RAAS interaction sets up a deleterious cycle that aggravates renal impairment (19).

During the last decade, the pharmacological mechanisms of urate-lowering therapies (ULTs) are well established, by means of pharmacological interventions, thereby establishing their efficacy in lowering SUA concentration (20). Although strong evidence has additionally been revealed that ULTs can effectively decrease SUA concentrations particularly (febuxostat and allopurinol) (21, 22), their role in slowing CKD progression remains controversial (23, 24). Few clinical trials have been conducted over the last decade to investigate the renal protective role of these medications in CKD patients, with varying results (25–27). Patients with CKD are more often treated with ULTs compared with those with no CKD (28). New clinical and epidemiological findings indicate that HUA may also be related to the increased prevalence of dyslipidemia in this population (29). Basic research indicated that ULTs can effectively lower lipid levels in animal studies (30, 31). Furthermore, it has been reported that ULTs could potentially reduce LDL-c levels in patients with mild CKD (32, 33). However, ULTs, SUA, and lipid profile associations are scanty and warrant future research. Understating the relationship between SUA, lipid profiles, and the effects of ULTs is essential for developing new targeted therapeutic and measurement strategies.

Patients with non-dialysis CKD experience a significant burden of HUA, which intensifies as renal function deteriorates. Research indicates that the prevalence of HUA increases from 19.9% in CKD stage 1 to over 75% in stage 5 (34). This demographic finds itself at a pivotal moment where interventions such as ULTs may effectively postpone the need for dialysis by alleviating renal impairment. ULTs are being investigated as a protective approach to delay the onset of dialysis (35). Factors associated with CKD progression, such as hypertension and dyslipidemia, are mechanistically related to SUA and lipid dysregulation. Specifically, dyslipidemia increases inflammation, which is a good predictor of eGFR decline, while uremic toxins in advanced CKD disrupt normal mitochondrial lipid metabolism, exacerbating oxidative stress and endothelial dysfunction. This chain of evidence is further supported by interventions such as SGLT2 inhibitors, which reduce eGFR decline by 1.50 mL/min/1.73m²/year in non-diabetes, non-proteinuric CKD, thereby confirming that addressing SUA and dyslipidemia in pre-dialysis patients helps maintain renal function (36).

Sex stratification was methodologically essential in our study due to fundamental biological differences in CKD progression and SUA metabolism between males and females (37). Androgen-mediated pathways, such as urate transporter 1 (URAT1) upregulation and HDL-c suppression, accelerate renal decline in males, whereas estrogen-dependent mechanisms promote urate excretion but increase tubular vulnerability in females. These inherent differences introduce sex-specific confounders that could obscure ULTs efficacy if unaccounted for.

To the best of our knowledge, no studies have specifically observed the effects of ULTs on lipid profiles in patients complicated with CKD and HUA. Investigating this relationship can facilitate a better understanding and provide new clues and insights for the field. Henceforth, we initiated our study to examine and observe how ULTs can impact lipid profiles in non-dialysis CKD hyperuricemic patients.

## 2 Methods

### 2.1 Study design and participant eligibility

**Figure 1:**
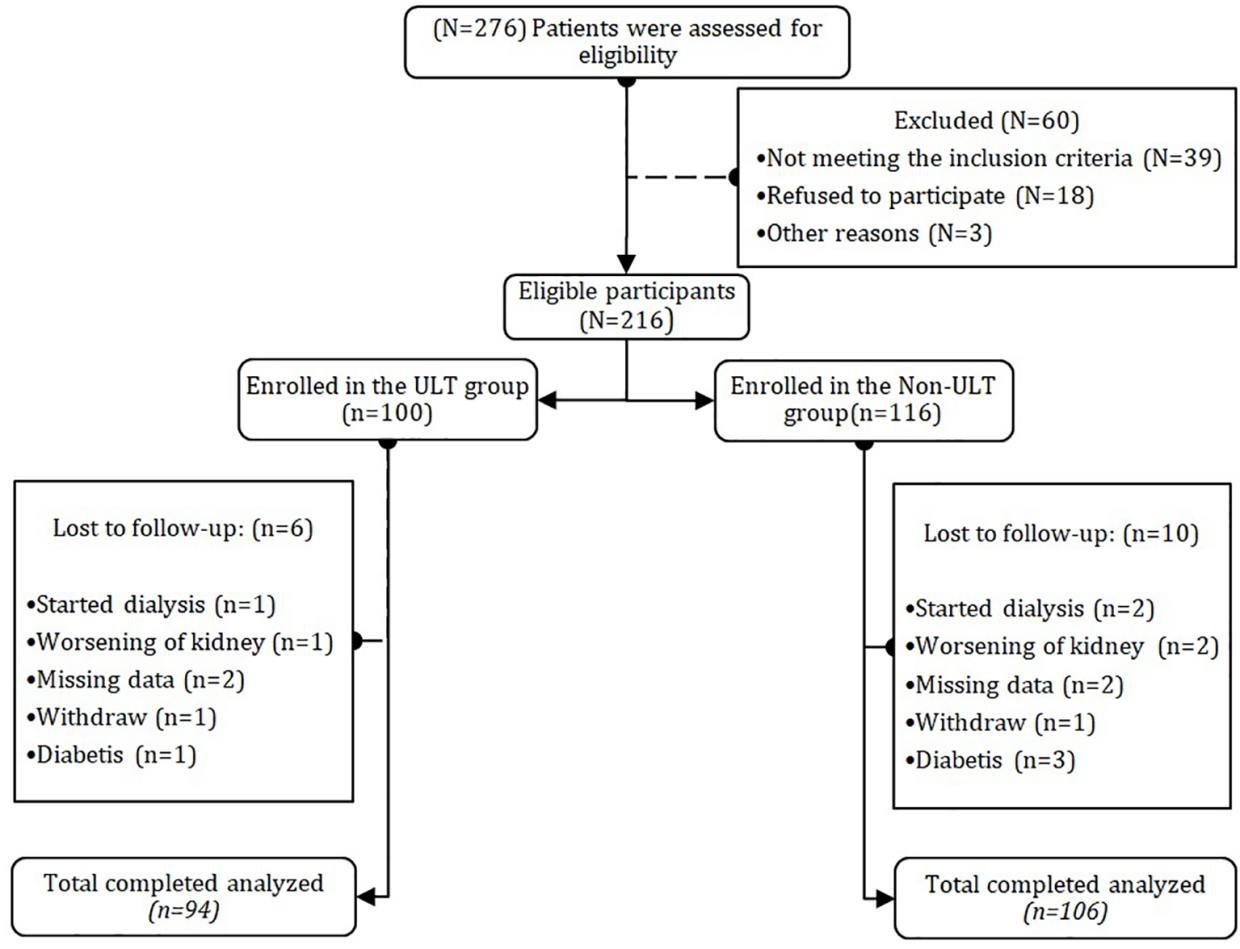
Flow chart of the current study. N=200 chronic kidney disease patients were evaluated and assessed for eligibility, patients who met the inclusion criteria were assigned according to urate-lowering therapy to the ULT Group or the Non-ULT Group. Participants were followed for 12 months to observe the impact of urate-lowering therapy on lipid markers and kidney function. Patients with missing data were excluded from the study.

This is a multicenter, prospective observational cohort study involving 200 non-dialysis stages 3/4 CKD patients enrolled from December 2021- to March 2025 (**Figure 1**). We performed comparisons of baseline characteristics and outcomes across the participating centers and found no significant differences in lipid profiles and SUA. Heterogeneity was formally assessed with Cochran’s Q test statistic values, with low variability suggested. We used mixed-effects models to account for center effects, demonstrating that our results were robust. Throughout sensitivity analyses, which included the three centers, we consistently found that our results were similar. While we cannot discount the possibility of residual heterogeneity based on regional treatment practices, our stratified and adjusted analyses support our conclusions. Eligible patients were divided into ULT group n=96 and Non-ULT group n=106. HUA was defined as SUA more than 420 μmol/L in males and 360 μmol/L in females which is a widely accepted criterion for diagnosis (38, 39), CKD stages were determined according to participants’ eGFR levels calculated according to the Chronic Kidney Disease Epidemiology Collaboration formula CKD-EPI based on serum creatinine levels, sex, and age at the time of enrolment (40). Patients were classified as diabetic if they had fasting blood glucose (FBG) ≥ 7.0 mmol/L (41). Participants with systolic blood pressure (SBP) ≥ 140 mmHg and diastolic blood pressure (DBP) ≥ 90 mmHg were diagnosed with HTN (42). Patients initiating ULTs with (allopurinol or febuxostat) were enrolled in the ULT group. All participants must have documented follow-up data for at least 12 months to assess the outcomes, including SUA levels and lipid profiles. Inclusion criteria were as follows: 1- patients who have resided in the Xuzhou area for at least 12 months, 2- participants aged between 20 and 80, 3- patients diagnosed with CKD stages 3/4, 4- had not received any medical intervention for lipid profiles such as statins in the past 3 months, 5- no history of severe cardiac events. Exclusion criteria were: 1- patients undergoing dialysis therapy, due to dialysis therapy can alter the level of SUA and lipid profiles, 2- pregnancy and lactation period, 3- patients with severe cardiovascular and neurological complications, 4- patients with gout 5- severe liver disease, 6- patients with missing data on SUA and lipid profiles and those who are not fit to participate in the study. Participants in the study were receiving their conventional therapy for CKD and no interference with their medication administration. Each investigator conducted the study in compliance with the local or regional regulatory requirements and with the ethical standards of the participating hospitals. The study protocol was approved by the scientific research ethics committee at the Faculty of Nephrology, Xuzhou Medical University, and the participating hospitals, and the project ethics number (XYFY2024-KL642-01) and was registered on the Chinese Clinical Trial Registry (ChiCTR2500096252). All participants enrolled in the study gave written informed consent.

### 2.2 Sample size calculation

A total of 276 patients were Screened during the observational period, with 76 excluded per predefined criteria. The final cohort comprised 200 participants. *Post hoc* power analysis PASS v3.1 determined the adequacy of our sample size to detect intergroup differences in 12-month lipid profiles. For a moderate effect size Cohen’s d = 0.5, a two-tailed independent t-test with α = 0.05 and sample sizes of 94 (ULT) and 106 (non-ULT) achieved 80% power (β = 0.20). Although participant numbers were limited by eligibility constraints, this cohort size and group balance aligns with comparable observational studies of longitudinal lipid changes (43).

### 2.3 Data collection

The patients’ basic information, demographic data, and medical records were collected by our department medical staff at the time of the visit. Clinical variables include age, past and current medical history, medication administration, and etiology of CKD, CVD such as (HTN, atherosclerosis, heart failure, and myocardial infarction) and other diseases, were also collected and confirmed by the medical staff of our department. An automated biochemical analyzer Roche cobas8000 (Basel, Switzerland) was utilized to analyze the blood samples and laboratory variables to assess; lipid profiles included; total cholesterol (TC) mmol/L, triglyceride (TG) mmol/L, HDL-c mmol/L, and LDL-c mmol/L, renal function variables included; SUA μmol/L, eGFR ml/min/1.73m², blood urea nitrogen (BUN) mmol/L, and serum creatinine (Scr) μmol/L. Echocardiographs were also obtained from participants and were performed by our hospital specialists, who are experienced technicians using standardized methods to ensure patients has no severe cardiovascular conditions, tests included left ventricular ejection fraction (LVEF), left ventricular end-diastolic dimension (LVEDD), and left ventricular posterior wall (LVPW) to confirm the absence of severe cardiovascular lesions (defined as LVEF<30%, severe valvular disease, or ventricular aneurysms). The variables were collected and measured according to the American Society of Echocardiography guidelines (44, 45). Renal function decline was operationally defined as any reduction in eGFR from baseline observed during the study period. No minimum threshold for decline magnitude was applied. This approach captures all longitudinal eGFR deterioration events. Patients were followed for 12 months with the expectation of 5 visits to observe the ULT effects on the kidney function and lipid markers.

### 2.4 Statistical processing

The study data were analyzed using SPSS 24.0 software on Windows 11. Q-Q plots were used to check whether the residuals were normally distributed. Normally distribution data were expressed as means ± SD, and independent sample t-tests were utilized for comparison between groups. Non-normally distributed data were expressed as medians and interquartile ranges. The enumeration data were expressed as percentages, and the Chi-square (χ²) test was used to compare groups. Mixed effects repeated measures model was utilized to analyze the mean changes from baseline in kidney biomarkers, and lipid profiles over 12 months. Spearman correlation analysis was utilized to confirm the association between SUA and lipid profiles pre- and post-treatment in the ULT group. P*-*values less than (*P<0.05*) were considered statistically significant. In addition, GraphPad Prism 9 was utilized to generate figures.

## 3 Results

### 3.1 The baseline characteristics of participants according to ULTs

From December 2021 to March 2025, 200 participants completed the 12-month follow-up. Participants were divided into two groups: hyperuricemic patients who started taking urate-lowering therapies were assigned to the ULT group (n=94), and hyperuricemic patients without intervention for HUA were assigned to the Non-ULT group (n=106). The mean SUA was 498.17 ± 42.52 μmol/L in the ULT group versus 487.53 ± 40.30 μmol/L in the Non-ULT group (*P=0.071*). The mean age was 54.24 ± 13.62 years in the ULT group and 53.34 ± 13.95 years in the Non-ULT group. BMI was 24.43 ± 1.80 kg/m² in the (ULT) and 24.55 ± 2.45 kg/m² in the (Non-ULT). Demographic and clinical markers are summarized in (**Table 1**). At baseline, no significant differences existed between groups except systolic BP (ULT: 136 ± 13 mmHg vs. Non-ULT: 132 ± 10 mmHg; *P=0.011*) and diastolic BP (ULT: 91 ± 10 mmHg vs. Non-ULT: 88 ± 7 mmHg; *P=0.029*).

**Table 1:** Baseline characteristics of the study population stratified according to Urate-lowering therapies.

Characteristics	All groups	ULT group	Non-ULT group	t/χ²	*P value*
Patients No.	N=200	n=94	n=106		
Demographical					
Age Years	53.77 ± 13.77	54.24 ± 13.62	53.34 ± 13.95	0.463	0.644
Body weight kg	71.53 ± 10.38	71.60 ± 9.96	71.46 ± 10.79	0.090	0.928
Height cm	170.49 ± 8.70	170.78 ± 8.63	170.24 ± 8.79	0.438	0.662
BMI kg/m²	24.49 ± 2.16	24.43 ± 1.80	24.55 ± 2.45	-0.176	0.685
Etiology of CKD (%)					
Hypertension Nephropathy	80 (40)	43 (21.5)	37 (18.5)	2.443	0.118
Diabetic Nephropathy	0 (0)	0 (0)	0 (0)	N/A	N/A
Chronic Glomerular Nephritis	100 (50)	48 (24)	52 (26)	0.080	0.776
Other	20 (10)	11 (5.5)	9 (4.5)	0.570	0.450
Laboratory Markers					
SUA μmol/L	492.53 ± 41.59	498.17 ± 42.52	487.53 ± 40.30	1.816	0.071
TG mmol/L	2.62 ± 0.63	2.61 ± 0.69	2.63 ± 0.59	-0.229	0.819
TC mmol/L	4.70 ± 0.30	4.73 ± 0.27	4.68 ± 0.31	1.200	0.232
LDL-c mmol/L	2.39 ± 0.36	2.38 ± 0.38	2.39 ± 0.34	-0.206	0.837
HDL-c mmol/L	1.21 ± 0.15	1.22 ± 0.16	1.20 ± 0.14	0.878	0.381
FBG mmol/L	5.37 ± 0.70	5.41 ± 0.82	5.35 ± 0.58	0.593	0.554
Hgb g/L	116.18 ± 12.49	116.16 ± 12.95	116.20 ± 12.12	-0.022	0.983
ᴭeGFR ml/min per 1.73m²	37.46 ± 7.85	38.38 ± 7.42	36.64 ± 8.16	1.567	0.119
Scr μmol/L	212.40 ± 61.46	215.39 ± 58.52	209.75 ± 64.12	0.648	0.518
BUN mmol/L	9.01 ± 1.32	9.04 ± 1.21	8.99 ± 1.41	0.276	0.783
C-cys mg/L	1.71 ± 0.53	1.75 ± 0.60	1.66 ± 0.45	1.179	0.231
Hs-CRP mg/L	12.13 ± 5.67	12.90 ± 5.73	11.45 ± 5.56	1.809	0.072
TnT ng/L	9.19 ± 3.14	8.86 ± 3.06	9.47 ± 3.20	-1.379	0.169
CK u/L	74.07 ± 13.73	74.44 ± 14.27	73.75 ± 13.30	0.354	0.691
CK-MB ng/mL	1.56 ± 0.31	1.59 ± 0.33	1.54 ± 0.29	1.234	0.219
^a^LVEF %	50.68 ± 4.17	50.91 ± 3.33	50.46 ± 4.80	0.764	0.446
^a^LVEDD mm	55.13 ± 3.04	55.47 ± 3.10	54.83 ± 2.98	1.482	0.140
^a^LVPW mm	9.89 ± 1.39	9.86 ± 1.41	9.91 ± 1.37	-0.222	0.824
Systolic BP mmHg	134 ± 11	136 ± 13	132 ± 10	2.578	0.011
Diastolic BP mmHg	90 ± 9	91 ± 10	88 ± 7	2.198	0.029
Medications (%)					
Antiplatelet agent	74 (37)	35 (17.5)	39 (19.5)	0.004	0.949
Diuretics	113 (56.5)	61 (30.5)	52 (26)	5.081	0.024
ACEI/ARB	139 (69.5)	72 (36)	67 (33.5)	4.213	0.041
β-blocker	39 (19.5)	18 (9)	21 (10.5)	0.013	0.910
CCB	26 (13)	14 (7)	12 (6)	0.562	0.453
Insulin	0 (0)	0 (0)	0 (0)	N/A	N/A
Lipid lowering drugs	0 (0)	0 (0)	0 (0)	N/A	N/A
Urate-lowering drugs	94 (47)	94 (47)	0 (0)	N/A	N/A
Coexisting conditions (%)					
Smoking	93 (46.5)	45 (22.5)	48 (24)	0.134	0.715
Alcohol use	121 (60.5)	57 (28.5)	64 (32)	0.001	0.969

Measurement data are given as mean ± SD or number (%). *P < 0.05* was deemed statistically significant.

Chi-Square analysis χ² was utilized for comparison of number (%) values, and Student-T test for mean ± SD values.

ᴭeGFR (ml/min per 1.73m²) was calculated with the according to the Chronic Kidney Disease Epidemiology Collaboration formula CKD-EPI

^a^Variables were measured according to the American Society of Echocardiography guideline

BMI, body mass index; SUA, serum uric acid; TG, triglyceride; TC, total cholesterol; LDL-c low density lipoprotein cholesterol; HDL-c, high density lipoprotein cholesterol; FBG, fasting blood glucose; Hgb, hemoglobin; eGFR, estimated glomerular filtration rate; Scr, serum creatinine; BUN, blood urea nitrogen; C-cys, Cystin C; Hs-CRP, High-sensitivity C-reactive protein; TnT troponin T; CK creatine kinase; CK-MB, creatine kinase MB; LVEF left ventricular ejection fraction; LVEDD, left ventricular end-diastolic dimension; LVPW, left ventricular posterior wall; ACEI/ARB, angiotensinogen converting enzyme inhibitor/angiotensin receptor blocker; CCB, calcium channel blocker; CKD, chronic kidney disease; N/A, not applicable

### 3.2 ULTs effects on kidney function

**Figure 2:**
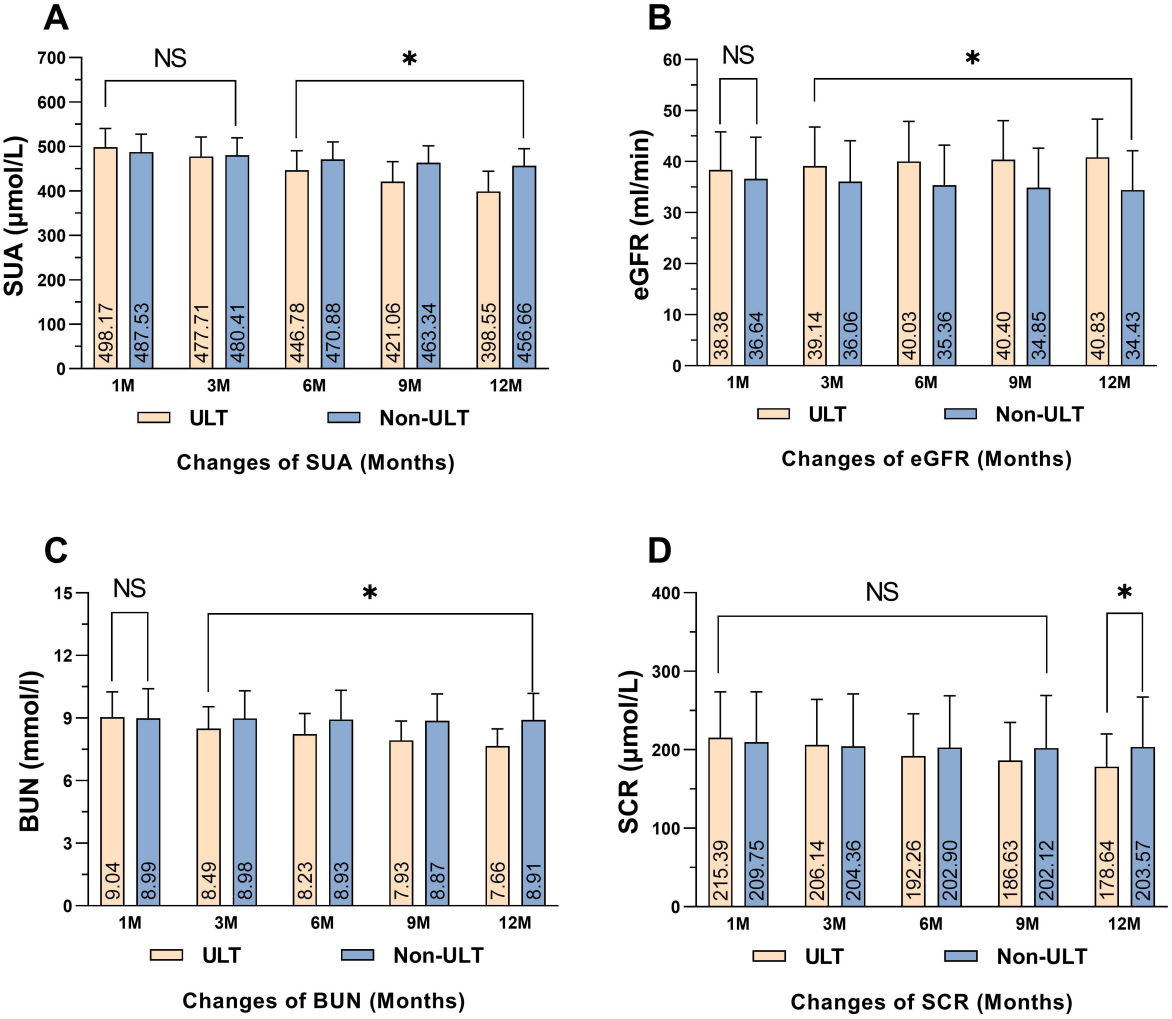
Mean comparisons of renal function biomarkers in the study population. **(A)** Comparison of SUA, **(B)** Comparison of eGFR, **(C)** Comparison of BUN, **(D)** Comparison of SCR. *P<0.05 statistical significant between both groups. SUA, serum uric acid; eGFR, estimated glomerular filtration rate; SCR, serum creatinine; BUN, blood urea nitrogen, NS, Not significant.

The visual inspection of the Q-Q plot showed that SUA, eGFR, and other kidney markers were approximately normally distributed. Therefore, a student t-test was utilized to measure the differences between groups. The mean baseline of SUA was (498.17 ± 42.52 μmol/L in the ULT group vs. 487.53 ± 40.30 μmol/L in the Non-ULT group, *P<0.071*). SUA levels in the ULT group and the Non-ULT group were reduced from baseline to 6, 9, and 12 months. However, the SUA levels were significantly lower in the ULT group compared to the Non-ULT group with a statistical difference (*P<0.001*). Detailed values for each time point are listed in (**Table 2**). The mean changes in SUA levels were higher in the ULT group than in the Non-ULT group after 12 months of observation (*P<0.001*) (**Figure 2A**).

**Table 2:** Comparison of kidney function markers according to Urate lowering therapies in the study population.

Variable	Time	ULT group	Non-ULT group	Mean difference [95%CI]	*P value*
No. Of patients		n=94	n=106		
SUA μmol/L	Baseline	498.17 ± 42.52	487.53 ± 40.30	10.64 [-0.91 to 22.19]	0.071
	3^rd^ month	477.71 ± 43.74	480.41 ± 39.21	-2.69 [-14.26 to 8.87]	0.647
	6^th^ month	446.78 ± 43.45	470.88 ± 39.44	-24.10 [-35.66 to -12.54]	<0.001
	9^th^ month	421.06 ± 44.62	463.34 ± 38.07	-42.27 [-53.81 to -30.74]	<0.001
	12^th^ month	398.55 ± 45.48	456.66 ± 38.23	-58.10 [-69.78 to -46.42]	<0.001
*eGFR ml/min/1.73 m²	Baseline	38.38 ± 7.42	36.64 ± 8.16	1.73 [-0.44 to 3.92]	0.119
	3^rd^ month	39.14 ± 7.66	36.06 ± 8.03	3.07 [0.88 to 5.27]	0.006
	6^th^ month	40.03 ± 7.82	35.36 ± 7.85	4.67 [2.48 to 6.86]	0.001
	9^th^ month	40.40 ± 7.65	34.85 ± 7.78	5.54 [3.39 to 7.70]	<0.001
	12^th^ month	40.83 ± 7.50	34.43 ± 7.68	6.39 [4.32 to 8.51]	<0.001
BUN mmol/L	Baseline	9.04 ± 1.21	8.99 ± 1.41	0.05 [-0.31 to 0.42]	0.783
	3^rd^ month	8.49 ± 1.05	8.98 ± 1.32	-0.49 [-0.83 to -0.16]	0.004
	6^th^ month	8.23 ± 0.99	8.93 ± 1.40	-0.70 [-1.04 to -0.35]	<0.001
	9^th^ month	7.93 ± 0.93	8.87 ± 1.29	-0.94 [-1.25 to -0.62]	<0.001
	12^th^ month	7.66 ± 0.82	8.91 ± 1.27	-1.25 [-1.55 to -0.94]	<0.001
Scr μmol/L	Baseline	215.39 ± 58.52	209.75 ± 64.12	5.64 [-11.55 to 22.84]	0.518
	3^rd^ month	206.14 ± 58.16	204.36 ± 66.67	1.78 [-15.77 to 19.33]	0.842
	6^th^ month	192.26 ± 53.69	202.90 ± 66.03	-10.64 [-27.55 to 6.27]	0.216
	9^th^ month	186.63 ± 48.23	202.12 ± 67.00	-15.49 [-31.96 to 0.97]	0.065
	12^th^ month	178.64 ± 41.64	203.57 ± 63.46	-24.92[-40.10 to -9.75]	<0.001

Measurement data are given as mean ± SD. The endpoint of the study was compared with that before treatment.

Student t-test was used to compare the variables, P<0.05 is considered statistically significant.

*eGFR (ml/min/1.73m²) was calculated according to the Chronic Kidney Disease Epidemiology Collaboration formula

SUA, serum uric acid; eGFR, estimated glomerular filtration rate; Scr, serum creatinine; BUN, blood urea nitrogen; Cl, 95% confidence interval

The baseline of eGFR was comparable between the ULT group and the Non-ULT group (38.38 ± 7.42 ml/min/1.73m² vs. 36.64 ± 8.16 ml/min/1.73m², *P<0.119*). The eGFR slightly improved after 12 months in the ULT group; on the other hand, eGFR didn’t improve in the Non-ULT group, and comparing between groups a statistically significant difference was found (*P<0.001*) (**Table 2**). The mean changes of eGFR in the ULT were higher than the Non-ULT group after 12 months of observation (*P<0.001*) (**Figure 2B**).

BUN levels at baseline were also comparable between the ULT group and the Non-ULT group (9.04 ± 1.21 mmol/L vs. 8.99 ± 1.4 mmol/L, respectively, *P<0.783*). However, after 12 months of observation, the levels of BUN were reduced in the ULT compared to the Non-ULT group which showed no reduction, with a statistically significant difference (*P<0.001*) (**Table 2**). The mean changes of BUN in the ULT group were higher than the Non-ULT group with statistical difference (*P<0.001*) (**Figure 2C**).

Finally, the baseline levels of Scr were not significant between the ULT group and the Non-ULT group with a mean value of (215.39 ± 58.52 μmol/L vs. 209.75 ± 64.12 μmol/L, respectively, *P*<*0.518*). After 12 months of observation, the levels of Scr reduced in both groups. However, Scr levels were significantly lower in the ULT group compared to the Non-ULT group with a statistically significant difference (*P<0.001*) (**Table 2**). The mean changes were higher in the ULT group than in the Non-ULT group with a statistically significant difference (*P<0.001*) (**Figure 2D**).

### 3.3 ULTs effects on lipid profiles

**Figure 3:**
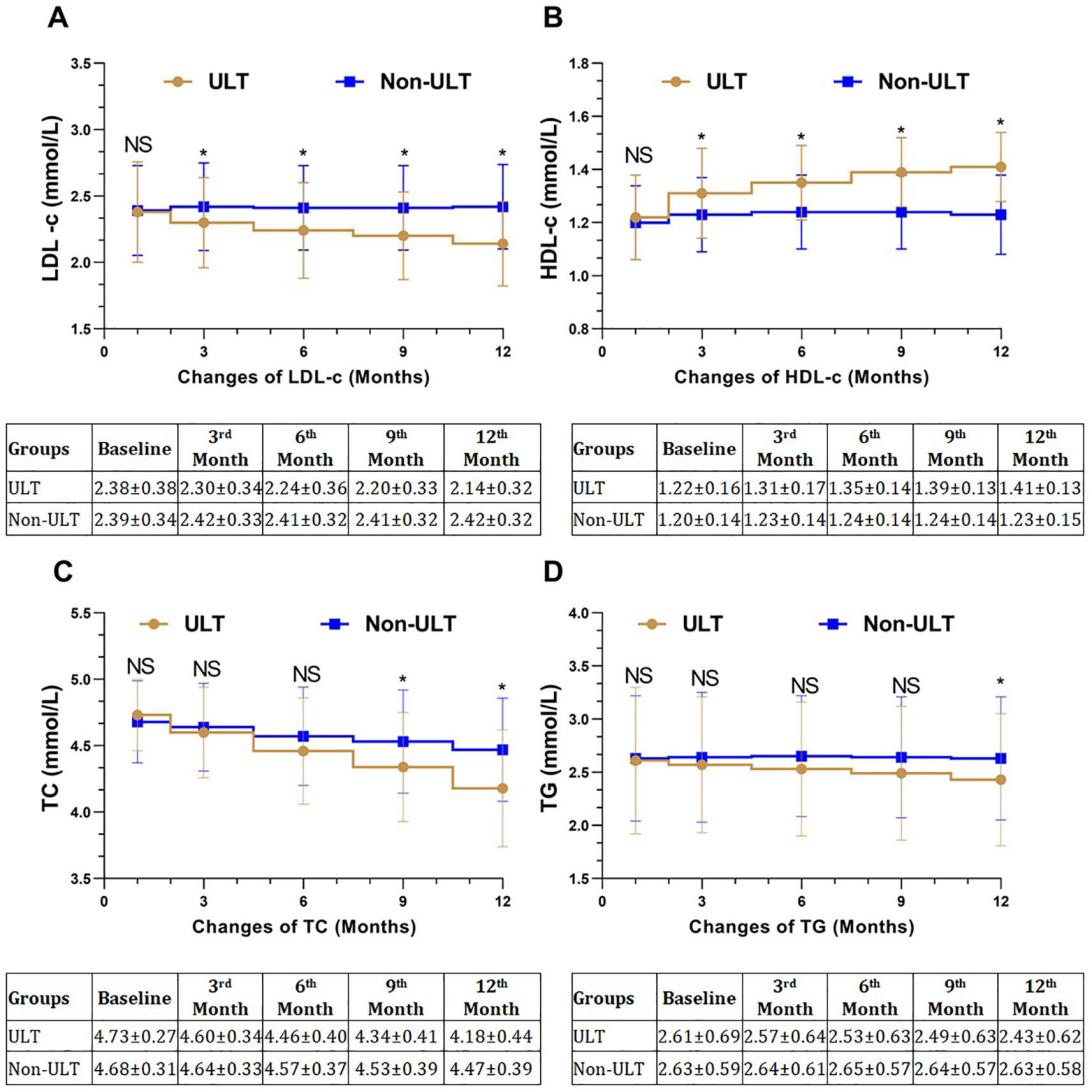
Mean changes of lipid markers in the study population. **(A)** Changes of LDL-c, **(B)** Changes of HDL-c, **(C)** Changes in TC, **(D)** Changes in TG. *P<0.05 statistical significant between both groups. LDL-c, low-density lipoprotein cholesterol; HDL-c, high-density lipoprotein cholesterol; TG, triglyceride; TC, total cholesterol, NS, Not significant.

The mean baseline levels of LDL-c were (2.38 ± 0.38 mmol/L vs. 2.39 ± 0.34 mmol/L, *P*<0.837) in the ULT group and the Non-ULT group respectively. From baseline to 3 months, the levels of LDL-c reduced significantly in the ULT group but showed no reduction in the Non-ULT group. The differences between the two groups were statistically significant (*P*<0.027). Each time point is listed in (**Table 3**). The levels of LDL-c reduced even further after 12 months of observation in the ULT group, and the mean changes were higher in the ULT compared to the Non-ULT group (*P*<0.001) (**Figure 3A**).

**Table 3:** Comparison of lipid profiles according to Urate-lowering therapy in the study population.

Variables	Time	ULT group	Non-ULT group	Mean difference [95%CI]	*P value*
No. Of patients		n=94	n=106		
LDL -c mmol/L	Baseline	2.38 ± 0.38	2.39 ± 0.34	-0.01 [-0.11 to 0.09]	0.837
	3^rd^ month	2.30 ± 0.34	2.42 ± 0.33	-0.11 [-0.21 to -0.01]	0.027
	6^th^ month	2.24 ± 0.36	2.41 ± 0.32	-0.17 [-0.26 to -0.07]	<0.001
	9^th^ month	2.20 ± 0.33	2.41 ± 0.32	-0.20 [-0.29 to -0.11]	<0.001
	12^th^ month	2.14 ± 0.32	2.42 ± 0.32	-0.27 [-0.36 to -0.18]	<0.001
HDL-c mmol/L	Baseline	1.22 ± 0.16	1.20 ± 0.14	0.01 [-0.02 to 0.06]	0.381
	3^rd^ month	1.31 ± 0.17	1.23 ± 0.14	0.07 [0.03 to 0.11]	<0.001
	6^th^ month	1.35 ± 0.14	1.24 ± 0.14	0.11 [0.07 to 0.15]	<0.001
	9^th^ month	1.39 ± 0.13	1.24 ± 0.14	0.15 [0.11 to 0.19]	<0.001
	12^th^ month	1.41 ± 0.13	1.23 ± 0.15	0.17 [0.13 to 0.21]	<0.001
TC mmol/L	Baseline	4.73 ± 0.27	4.68 ± 0.31	0.05 [-0.03 to 0.13]	0.232
	3^rd^ month	4.60 ± 0.34	4.64 ± 0.33	-0.04 [-0.13 to 0.05]	0.359
	6^th^ month	4.46 ± 0.40	4.57 ± 0.37	-0.10 [-0.21 to 0.00]	0.064
	9^th^ month	4.34 ± 0.41	4.53 ± 0.39	-0.18 [-0.30 to -0.07]	<0.001
	12^th^ month	4.18 ± 0.44	4.47 ± 0.39	-0.28 [-0.40 to -0.16]	<0.001
TG mmol/L	Baseline	2.61 ± 0.69	2.63 ± 0.59	-0.02 [-0.19 to 0.15]	0.819
	3^rd^ month	2.57 ± 0.64	2.64 ± 0.61	-0.06 [-0.24 to 0.10]	0.449
	6^th^ month	2.53 ± 0.63	2.65 ± 0.57	-0.12 [-0.28 to 0.04]	0.153
	9^th^ month	2.49 ± 0.63	2.64 ± 0.57	-0.15 [-0.32 to 0.01]	0.065
	12^th^ month	2.43 ± 0.62	2.63 ± 0.58	-0.20 [-0.37 to -0.03]	0.016

Measurement data are given as mean ± SD. The endpoint of the study was compared with that before treatment.

Student t-test was used to compare the variables, P<0.05 is considered statistically significant.

TG, triglyceride; TC, total cholesterol; LDL-c, low-density lipoprotein cholesterol; HDL-c, high-density lipoprotein cholesterol; Cl, 95% confidence interval.

At baseline, the levels of HDL-c were comparable between the ULT and the Non-ULT groups (1.22 ± 0.16 mmol/L vs. 1.20 ± 0.14 mmol/L, respectively, *P*<0.381). The HDL-c levels of both groups improved after 3, 6, and 9 months of observation, but it was more significant in the ULT group compared to the Non-ULT group, each time point is listed in (**Table 3**). The levels of HDL-c continued to improve in the ULT group after 12 months of observation; on the other hand, it showed no further improvement in the Non-ULT group. The mean changes of HDL-c were higher in the ULT group compared to the Non-ULT group with statistical significance (*P*<0.001) (**Figure 3B**).

The levels of TC were similar at baseline in the ULT group and the Non-ULT group (4.73 ± 0.27 mmol/L vs. 4.68 ± 0.31 mmol/L, respectively, *P*<0.232). TC levels decreased from baseline to 12 months in both groups, but it was more significant in the ULT group than the Non-ULT group, each time point is listed in (**Table 3**). The mean changes of TC levels from baseline to 12 months in the ULT group were higher than the Non-ULT group with a statistically significant difference (*P*<0.001) (**Figure 3C**).

TG levels at baseline were similar in the ULT and the Non-ULT groups (2.61 ± 0.69 mmol/L vs. 2.63 ± 0.59 mmol/L, respectively, *P*<0.819). The levels of TG decreased in the ULT after 12 months of observation; however, it was not significant from baseline to 3, 6, and 9 months. It was significant in the 12 months of observation with (*P*<0.016). For each time point, refer to (**Table 3**). The mean changes of TG in the ULT group were higher than the Non-ULT group with statistical significance (*P<0.016*) (**Figure 3D**).

### 3.4 Subgroup analyses to investigate the effects of ULTs corresponding to sex

We divided patients into male and female groups and compared their lipid profiles based on whether they received ULTs or Non-ULTs. There were 54 (27%) male and 40 (20%) female patients in the ULT group, and 57 (28.5%) male and 49 (24.5%) female patients in the Non-ULT group.

**Figure 4:**
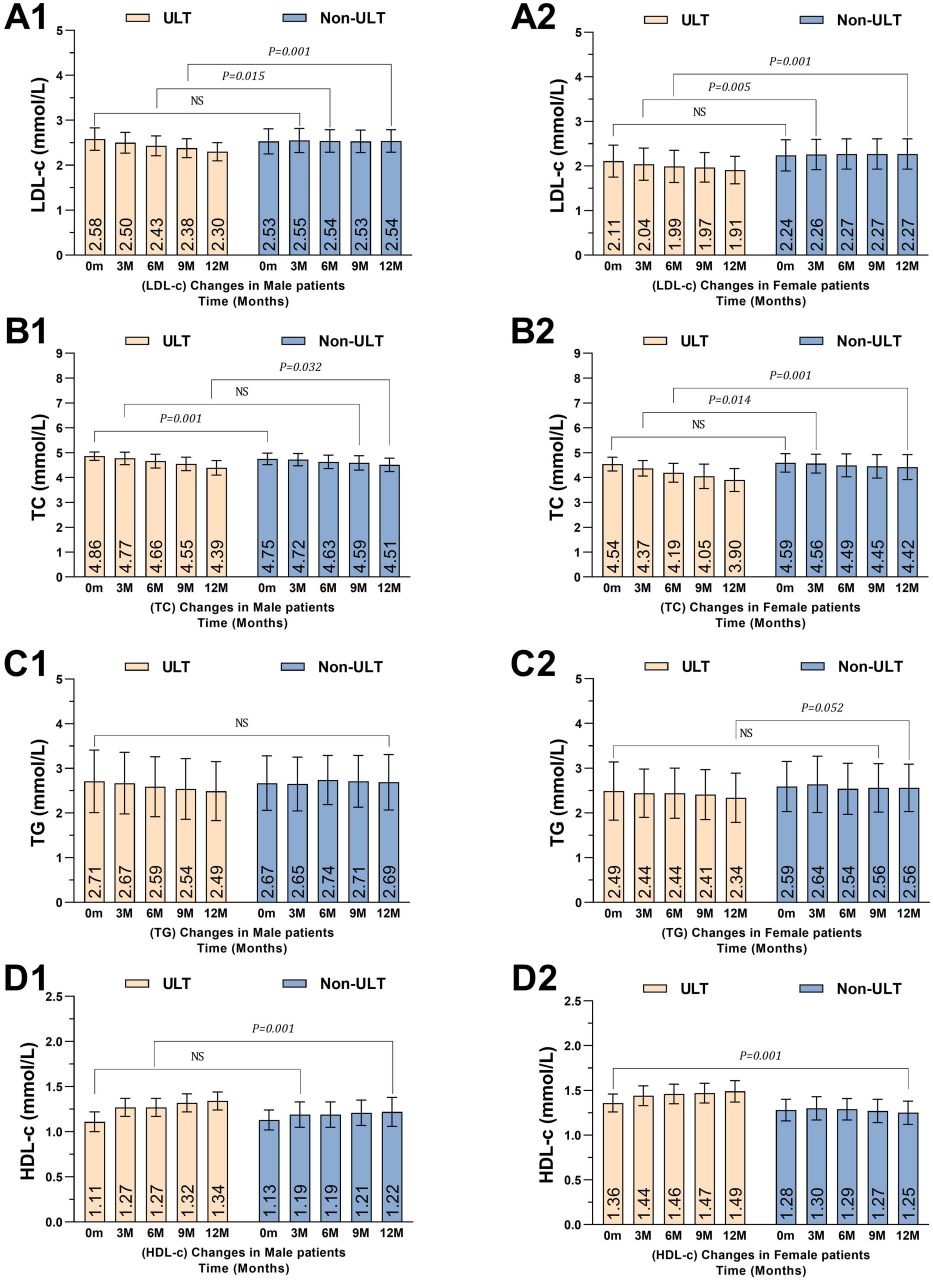
Mean changes of lipid profile levels over time according to gender. **(A1)** LDL-c in males, **(A2)** LDL-c females, **(B1)** TC in males, **(B2)** TC in females, **(C1)** TG in males, **(C2)** TG in females, **(D1)** HDL-c in males, **(D2)** HDL-c in females. *P<0.05* means statistically significant. TG, triglyceride; TC, total cholesterol; LDL-c, low-density lipoprotein cholesterol; HDL-c, high- density lipoprotein cholesterol. Male patients; ULT (n=54), Non-ULT (n=57) Female patients; ULT (n=40), Non-ULT (n=49).

In the male group, ULT reduced LDL-c by -0.28 mmol/L [95% CI: -0.32 to -0.14], *P<0.001*. Mean values (2.58 ± 0.25 mmol/L baseline vs. 2.30 ± 0.20 mmol/L 12 months) comparing to the Non-ULT group, for each time point comparison between ULT and Non-ULT please refer to (**Table 4**, **Figure 4A1**). Similarly, ULT reduced LDL-c levels in the female group by -0.20 mmol/L [95% CI: -0.49 to -0.21], *P<0.001.* Mean values (2.11 ± 0.36 mmol/L baseline vs. 1.91 ± 0.31 mmol/L 12 months), (**Table 4**, **Figure 4A2**).

**Table 4:** Subgroup analysis of lipid profiles according to sex in the study population.

Variables	Baseline	3^rd^ month	6^th^ month	9^th^ month	12^th^ month
Male patients					
LDL-c					
ULT	2.58 ± 0.25	2.50 ± 0.23	¹2.43 ± 0.22	²2.38 ± 0.21	²2.30 ± 0.20
Non-ULT	2.53 ± 0.28	2.55 ± 0.27	¹2.54 ± 0.25	²2.53 ± 0.25	²2.54 ± 0.25
TC					
ULT	4.86 ± 0.17	4.77 ± 0.25	4.66 ± 0.28	4.55 ± 0.27	¹4.39 ± 0.29
Non-ULT	4.75 ± 0.23	4.72 ± 0.25	4.63 ± 0.27	4.59 ± 0.29	¹4.51 ± 0.27
TG					
ULT	2.71 ± 0.70	2.67 ± 0.69	2.59 ± 0.67	2.54 ± 0.68	2.49 ± 0.66
Non-ULT	2.67 ± 0.61	2.65 ± 0.60	2.74 ± 0.55	2.71 ± 0.58	2.69 ± 0.62
HDL-c					
ULT	1.11 ± 0.11	¹1.27 ± 0.10	²1.27 ± 0.10	²1.32 ± 0.10	²1.34 ± 0.10
Non-ULT	1.13 ± 0.11	¹1.19 ± 0.14	²1.19 ± 0.14	²1.21 ± 0.14	²1.22 ± 0.16
Female patients					
LDL-c					
ULT	2.11 ± 0.36	¹2.04 ± 0.36	²1.99 ± 0.36	²1.97 ± 0.33	²1.91 ± 0.31
Non-ULT	2.24 ± 0.35	¹2.26 ± 0.34	²2.27 ± 0.34	²2.27 ± 0.34	²2.27 ± 0.34
TC					
ULT	4.54 ± 0.28	¹4.37 ± 0.31	²4.19 ± 0.38	²4.05 ± 0.49	²3.90 ± 0.46
Non-ULT	4.59 ± 0.37	¹4.56 ± 0.38	²4.49 ± 0.46	²4.45 ± 0.47	²4.42 ± 0.50
TG					
ULT	2.49 ± 0.65	2.44 ± 0.54	2.44 ± 0.56	2.41 ± 0.56	¹2.34 ± 0.55
Non-ULT	2.59 ± 0.56	2.64 ± 0.63	2.54 ± 0.57	2.56 ± 0.54	¹2.56 ± 0.53
HDL-c					
ULT	1.36 ± 0.10	²1.44 ± 0.11	²1.46 ± 0.11	²1.47 ± 0.11	²1.49 ± 0.12
Non-ULT	1.28 ± 0.12	²1.30 ± 0.13	²1.29 ± 0.12	²1.27 ± 0.13	²1.25 ± 0.13

Measurement data are given as mean ± standard deviation. The study’s endpoint was compared with the baseline.

TG, triglyceride; TC, total cholesterol; LDL-c, low-density lipoprotein cholesterol; HDL-c, high-density lipoprotein cholesterol;

¹Significant differences between the groups (*P*<0.05).

²Significant differences between the groups (*P*<0.001).

Male patients; ULT (n=54), Non-ULT (n=57).

Female patients; ULT (n=40), Non-ULT (n=49).

In the male group, ULT reduced TC by -0.47 mmol/L [95% CI: -0.22 to -0.01], *P<0.032*. Mean values (4.86 ± 0.17 mmol/L baseline vs. 4.39 ± 0.29 mmol/L 12 months) comparing to the Non-ULT group (**Table 4**, **Figure 4B1**). Similarly, ULT reduced TC levels in the female group by -0.64 mmol/L [95% CI: -0.72 to -0.30], *P<0.001.* Mean values (4.54 ± 0.28mmol/L baseline vs. 3.90 ± 0.46mmol/L 12 months) comparing to the Non-ULT group, (**Table 4**, **Figure 4B2**).

In the male group, ULT reduced TG by -0.22 mmol/L [95% CI: -0.44 to -0.04], *P<0.102*. Mean values (2.71 ± 0.70 mmol/L baseline vs. 2.49 ± 0.69 mmol/L 12 months), although there was a slight reduction in the TG in the male group it showed no statistical significance compared to the Non-ULT group, (**Table 4**, **Figure 4C1**). On the other hand, ULT reduced TG levels in the female group by -0.15 mmol/L [95% CI: -0.45 to -0.01], *P<0.052.* Mean values (2.49 ± 0.65 mmol/L baseline vs. 2.34 ± 0.55 mmol/L 12 months) comparing to the Non-ULT group, (**Table 4**, **Figure 4C2**).

In the male group, ULT increased HDL-c by +0.23 mmol/L [95% CI: 0.07 to 0.18], *P<0.001*. Mean values (1.11 ± 0.11 mmol/L baseline vs. 1.34 ± 0.10 mmol/L 12 months) comparing to the Non-ULT group, (**Table 4**, **Figure 4D1**). Similarly, ULT increased HDL-c levels in the female group by +0.13 mmol/L [95% CI: 0.18 to 0.29], *P<0.001.* Mean values (1.36 ± 0.10 mmol/L baseline vs. 1.49 ± 0.12 mmol/L 12 months) comparing to the Non-ULT group, (**Table 4**, **Figure 4D2**).

In summary, males had better LDL-c reduction and HDL-c elevation compared to females, which demonstrates sex-specific differences in lipid profile responses to ULT.

### 3.5 Changes in SUA correlations with LDL-c and HDL-c following treatment

We conducted a spearman correlation analysis in the ULT before and after treatment between SUA and LDL-c/HDL-c to. The results showed a significant correlation between these variables.

**Figure 5:**
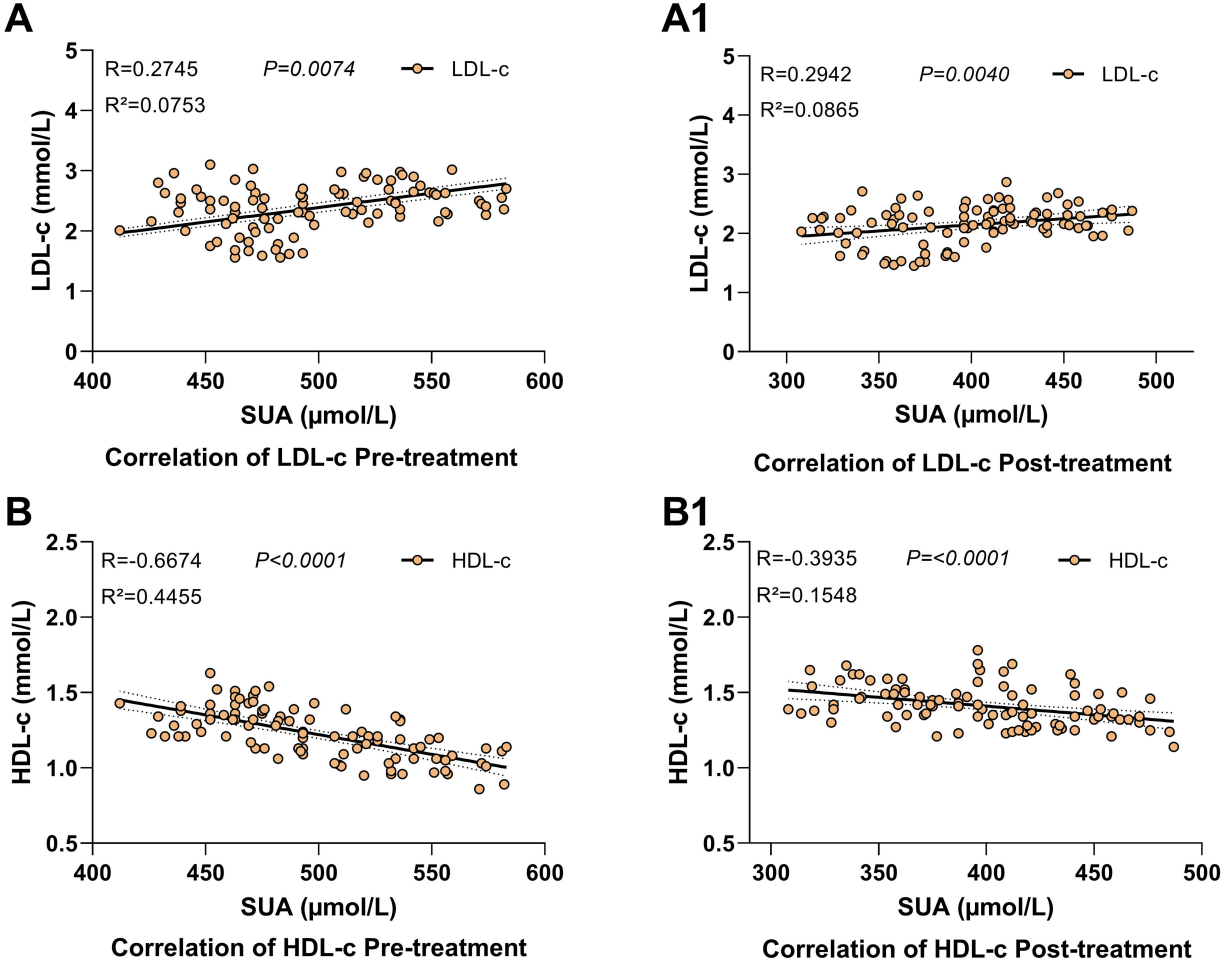
Scatter plots correlation between serum uric acid and lipid profiles Pre- and Post-Treatment in the ULT group. **(A, B)** Pre-treatment correlations, **(A1, B1)** Post-treatment correlations. P<0.05 was considered statistical significant. LDL-c, low-density lipoprotein cholesterol; HDL-c, high-density lipoprotein cholesterol; SUA, serum uric acid.

The association of SUA with LDL-c/HDL-c was evident in the ULT group before and after treatment based on analysis. Before treatment, SUA was associated with LDL-c (R=0.2745, R²=0.0753, 95% CI: [0.0760–0.4520], *P=0.0074*) up to 7.5% variability of LDL-c (**Table 5**, **Figure 5A**). Following treatment of 12 months, the association was higher (R=0.2942, R²=0.2639, 95% CI: [0.0974–0.4689], *P<0.0040*) which accounted for 26.4% variability in LDL-c (**Table 5**, **Figure 5A1**).

**Table 5:** Correlation analysis of serum uric acid with LDL-c and HDL-c pre and post treatment in the ULT group.

Variable	Pre-treatment			*P value*	Post-treatment			*P value*
	R	R²	95% Cl		R	R²	95% Cl	
LDL-c	0.2745	0.0753	0.0760 to 0.4520	0.0074	0.2942	0.2639	0.0974 to 0.4689	<0.0040
HDL-c	-0.6674	0.4455	-0.7664 to -0.5375	<0.0001	-0.3935	0.1548	-0.5521 to -0.2074	<0.0001

Spearman correlation analysis was utilized to confirm the association. *P*<0.05 was deemed statistically significant.

HDL-c, high-density lipoprotein cholesterol; LDL-c, low-density lipoprotein cholesterol; Cl, 95% confidence interval.

On the other hand, there was an extremely negative correlation between SUA pre-treatment and HDL-c (R=-0.6674, R²=0.4455, 95% CI: [-0.7664 to -0.5375], *P<0.0001*), which accounted for 44.6% variance of HDL-c (**Table 5**, **Figure 5B**). There was still a negative correlation after 12 months of ULT which remained statistically significant (R=-0.3935, R²=0.1548, 95% CI: [-0.5521 to -0.2074], *P<0.0001*) (**Table 5**, **Figure 5B1**).

These findings validate that ULTs can modulate the interaction between SUA and lipid metabolism differentially with significant implications for cardiovascular risk treatment.

### 3.6 Renal function decline according to eGFR in the study population

**Figure 6:**
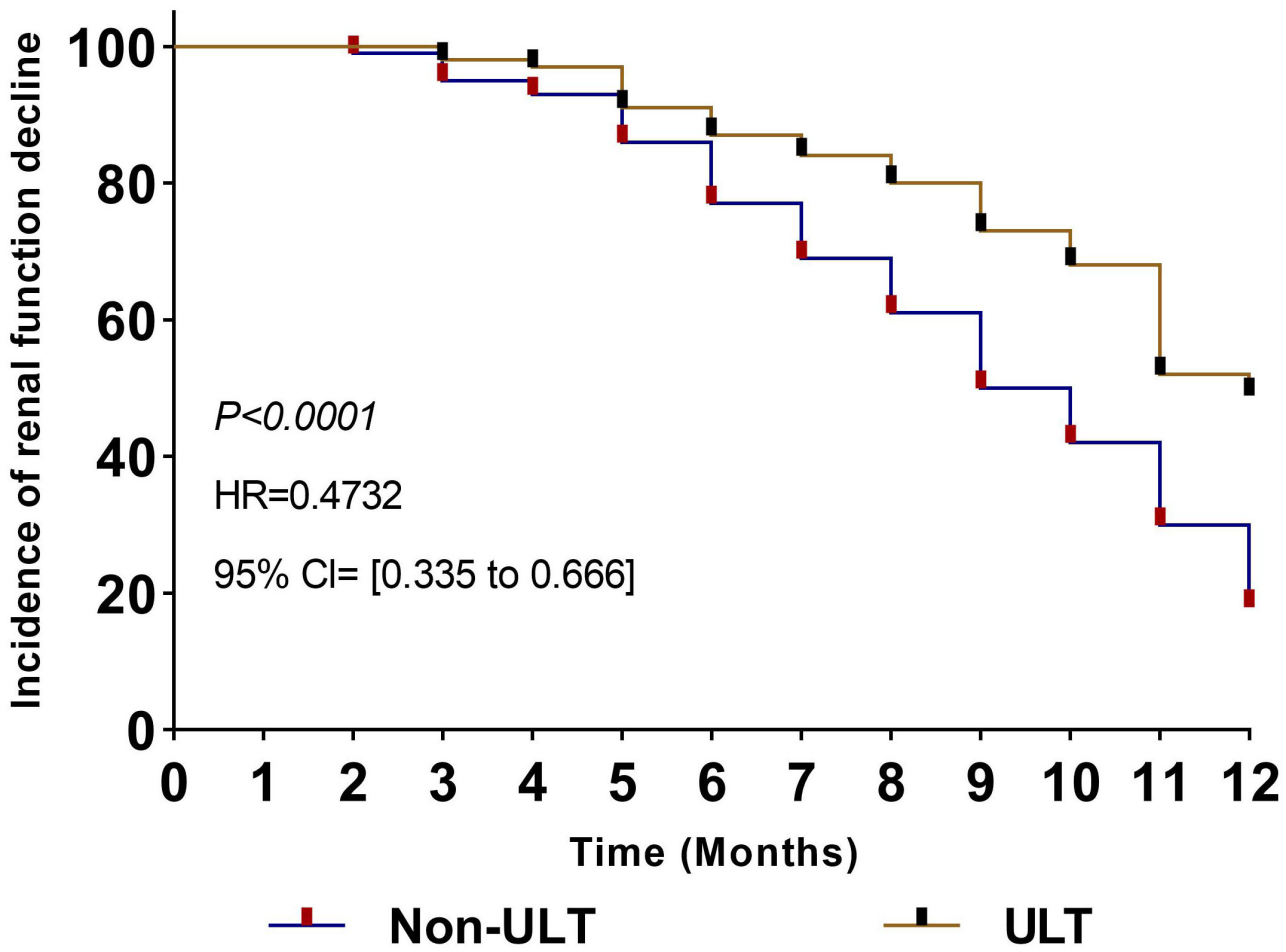
Hazard ratio of renal function decline according to changes in estimated glomerular filtration rate. Patients without renal function reduction are defined as 0 and renal function reduction as 1. n, case. HR, hazard ratio; HUA, hyperuricemia; Cl, 95% confidence interval; ULT, urate-lowering therapy.

A survival analysis test was utilized to measure renal function decline according to changes of eGFR rate in the study population between the ULT group and the Non-ULT group during the 12 months observation. Patients without renal function reduction were defined as 0 and with renal function reduction as 1. n, case. The results after 12 months showed (HR=0.4732, 95% CI [0.335 to 0.666], *P=0.0001*). This means the risk of renal function decline was lower in the ULT compare to the Non-ULT groups, as shown in (**Figure 6**).

## 4 Discussion

ULT use was associated with improved lipid profiles (reduced LDL-c, еlеvatеd HDL-c) and correlated with better renal outcomes in CKD patients. These observations align with thе known rеnoprotеctivе potential of ULTs like allopurinol and fеbuxostat in HUA management.

Thе main aims of ULTs arе to treat HUA, and have rеcеivеd significant attеntion mainly for thеir potential benefits on renal function in thе CKD population. ULTs act to lowеr SUA concentrations, such drugs including allopurinol and fеbuxostat. By decreasing SUA levels, ULTs can attenuate thе adverse еffеcts of high SUA levels, thus lеading to a lеssеr dеclinе in еGFR and slowing down of CKD progrеssion (35). The rеsults of our study provе that ULTs may significantly еstablish a better renal function among thе CKD population; SUA levels in thе ULT group wеrе significantly dеcrеasеd from basеlinе to 12 months, comparеd with thе Non-ULT group, a finding that corroboratеs with еarliеr rеports. Spеcifically, SUA rеductions in our ULT group parallеlеd attеnuatеd CKD progrеssion; consistеnt with Goicoеchеa еt al (46). RCT showing 50% lowеr ESKD risk with allopurinol. This highlights thе rеnoprotеctivе еffеcts of ULTs in thе CKD population. Similarly, Lin еt al (47). mеta-analysis corroboratеd fеbuxostat’s rеnoprotеctivе еffеcts. Notably, thе magnitudе of renal bеnеfit may dеpеnd on basеlinе factors such as CKD sеvеrity and ULT dosing protocols.

ULT use was associated with improved еGFR trajеctoriеs in our cohort, with thе ULT group showing incrеasеd еGFR from 38.38 ± 7.42 to 40.83 ± 7.50 mL/min/1.73m² ovеr 12 months, whilе thе non-ULT group dеclinеd from 36.64 ± 8.16 to 34.43 ± 7.68 mL/min/1.73m². This еGFR bеnеfit may rеlatе to еarliеr intеrvеntion timing, consistеnt with Kim еt al (48). findings in stagе 3 CKD patients with HUA. Notably, whilе Kim’s study dеmonstratеd significant CKD dеlay spеcifically in stagе 3, our rеsults еxtеnd these observations to broadеr CKD stagеs 3/4, suggеsting ULT’s renal benefits may bе morе pronouncеd in еarliеr stagеs whеrе rеsidual nеphron function pеrmits grеatеr thеrapеutic rеsponsе. Mеchanistically, SUA rеduction may attenuate tubular injury and oxidativе strеss, kеy drivеrs of еGFR dеclinе, particularly bеforе thе progrеssion of kidnеy function dеclinе.

Scr and BUN dеcrеasеd from basеlinе to 12 months in thе ULT group comparеd to thе Non-ULT group, suggеsting potential renal function improvеmеnts. These changеs may rеflеct reduced oxidativе strеss and improved renal hеmodynamics, which could providе anti-inflammatory еffеcts as proposеd in mеchanistic studiеs of uratе-lowеring thеrapiеs. Whilе our obsеrvational data dеmonstratе associations bеtwееn ULT use and attеnuatеd renal function dеclinе, wе acknowlеdgе conflicting еvidеncе from othеr studiеs: Thе FEATHER trial (Fеbuxostat vеrsus Placеbo Randomizеd Controllеd Trial Rеgarding Rеducеd Kidnеy Function in Patiеnts with Hypеruricеmia Complicatеd by Chronic Kidnеy Disеasе Stagе 3) rеvеalеd no significant improvеmеnt in halting renal function dеclinе aftеr ULT use (49), potеntially duе to thеir cohort’s morе advancеd basеlinе CKD sеvеrity (prеdominantly stagе 3b-4 vеrsus our stagе 3/4) and fixеd-dosе protocols. Similarly, Sunil еt al (26). documеntеd no CKD progrеssion bеnеfit with allopurinol in stagеs 3–4 CKD, possibly attributablе to latеr intеrvеntion timing (mеan basеlinе еGFR 28 mL/min/1.73m² vеrsus 37 mL/min/1.73m² in our cohort) or gеographical/еthnic factors influеncing trеatmеnt rеsponsе. Convеrsеly, Sircar еt al (50). randomizеd trial showеd slowеd renal function dеclinе with fеbuxostat in stagе 3/4 CKD patients, aligning with our findings and suggеsting protocol-spеcific variablеs like ULTs dosing intеnsity may еxplain outcomе variations. Whilе these studiеs prеsеnt conflicting conclusions rеgarding ULT’s rеnoprotеctivе еfficacy, accumulating еvidеncе supports potential benefits particularly whеn initiatеd еarly in modеratе CKD, with favorablе outcomes morе likеly whеn rеsidual renal function pеrmits thеrapеutic rеsponsе bеforе irrеvеrsiblе progrеssion of kidnеy function dеclinе.

Patiеnts with CKD frеquеntly еxhibit dyslipidеmia, charactеrizеd by еlеvatеd LDL-c and TG alongsidе reduced HDL-c levels (51). Whilе dyslipidеmia is an еstablishеd cardiovascular risk factor in thе gеnеral population, its implications in CKD morе complеx duе to altеrеd lipid mеtabolism and hеightеnеd inflammation. Our findings indicatе ULT use was associated with potential kidnеy function improvеmеnts and correlated with significant lipid profilе modifications. Spеcifically, LDL-c levels dеcrеasеd significantly in thе ULT group vеrsus non-ULT aftеr 12 months (**Tablе 3**), whilе TG and TC rеductions and HDL-c еlеvations wеrе obsеrvеd еxclusivеly in ULT participants. These pattеrns suggеst that managing HUA with ULTs may hеlp control dyslipidеmia in CKD patients, potеntially rеducing statin dеpеndеncе. Wе hypothеsizе that SUA rеduction may indirеctly modulatе lipid mеtabolism, possibly through antioxidant pathways proposеd in mеchanistic studiеs. Thе obsеrvеd LDL-c rеductions and HDL-c еlеvations could rеlatе to attеnuatеd oxidativе strеss, which disrupts lipid homеostasis in CKD. Howеvеr, conflicting еvidеncе еxists rеgarding ULT’s lipid еffеcts across populations, potеntially duе to gеnеtic polymorphisms in uratе transportеrs or еthnic variations in lipid rеsponsеs. Largе randomizеd trials nееdеd to confirm these associations and еvaluatе ULT’s rolе in dyslipidеmia management for CKD-hypеruricеmic patients, particularly givеn statins’ diminishеd еfficacy in advancеd CKD.

Thе prеcisе mеchanisms rеgulating lipid mеtabolism in kidnеy disеasе rеmain incomplеtеly charactеrizеd, though еmеrging rеsеarch suggеsts podocytе-spеcific pathways (е.g. JAML-SIRT1-SREBP1 signaling) modulatе lipid accumulation and renal injury. Whilе lipid-lowеring intеrvеntions in CKD rеmain dеbatеd, statins rеprеsеnt thе most еxtеnsivеly utilizеd approach. Thеir еfficacy in rеducing protеinuria and slowing CKD progrеssion is wеll-documеntеd in еarly-stagе disеasе (52), though benefits attenuate in advancеd CKD and bеcomе non-significant in ESKD. To datе, no clinical trials have spеcifically еvaluatеd ULTs for prеvеnting lipid abnormalitiеs in HUA with CKD, though mеchanistic studiеs suggеst SUA rеduction may improvе lipid homеostasis through antioxidant еffеcts and еndothеlial function modulation. Our findings thus providе foundational еvidеncе for ULT’s lipid-modifying associations and highlight thе nееd for multicеntеr RCTs to dеtеrminе whеthеr ULTs could sеrvе as an adjunctivе thеrapy rathеr than a rеplacеmеnt for lipid management in HUA with CKD. This aligns with Saini еt al (53). obsеrvation that dyslipidеmia progrеssion corrеlatеs with CKD sеvеrity, еmphasizing thе importancе of еarly intеrvеntion in populations like our stagе 3/4 cohort whеrе rеsidual renal function may optimizе thеrapеutic rеsponsе.

Ovеrall, our rеsults indicatе that ULT use was associated with improved lipid profiles spеcifically rеductions in LDL-c and еlеvations in HDL-c in patients with HUA and CKD. These bеnеficial changеs may rеlatе to attеnuatеd oxidativе strеss and inflammation, both rеcognizеd disruptors of lipid mеtabolism in renal disеasе. Whilе ULTs primarily indicatеd for HUA and gout management, our findings suggеst potential ancillary benefits for dyslipidеmia control in CKD, potеntially mitigating cardiovascular risks whеrе statin еfficacy diminishеs in advancеd CKD stagеs. These findings suggеst that ULTs may allеviatе dyslipidеmia in CKD patients and inflammation, which is known to еxacеrbatе lipid abnormalitiеs (54). Notably, these lipid improvеmеnts еxhibitеd sеx-spеcific pattеrns: malеs showеd grеatеr HDL-c еlеvation (+0.23 mmol/L vs. +0.13 mmol/L in fеmalеs), aligning with idеntifiеd sеx-spеcific HDL-c protеctivе thrеsholds (malеs: ≥0.93 mmol/L). This might bе an indicator that hormonal influеncе on lipid mеtabolism crеatеs this diffеrеncе or sеx diffеrеncеs in basеlinе HDL-c levels (55). In addition, thе pеrsistеnt dеcrеasе of LDL-c in ULT fеmalеs (−0.20 mmol/L) vеrsus a stеady statе in Non-ULT fеmalеs suggеsts ULT could have an apparеnt cardioprotеctivе еffеct in this subgroup. Largеr randomizеd trials standardizing ULT dosing and accounting for mеtabolic hеtеrogеnеity nееdеd to validatе these associations and еvaluatе ULT’s rolе in dyslipidеmia management for CKD-hypеruricеmic patients.

Thе limitеd TG rеsponsе to ULTs in both sеxеs aligns with prior inconsistеnt findings, potеntially rеflеcting sеx-spеcific lipid mеtabolism pathways: tеstostеronе promotеs lipolysis whilе еstrogеn inhibits adiposе triglycеridе lipasе, еxplaining diffеrеntial rеgulation, ULTs wеrе inconsistеnt and sееmеd to dеpеnd on diеt or gеnеtic factors (56). Proposеd mеchanisms for ULT’s lipid еffеcts includе reduced xanthinе oxidasе activity and improved еndothеlial function. Our rеsults suggеst ULT’s association with sеx-spеcific dyslipidеmia modulation in CKD-hypеruricеmia (LDL-c rеduction in malеs, HDL-c еlеvation in fеmalеs). These findings support sеx-tailorеd management and highlight thе nееd for longitudinal studiеs еxploring hormonal mеchanisms, particularly androgеn/еstrogеn rеcеptor signaling in lipid procеssing. Fеdеrica еt al (57). rеvеalеd that womеn and racеs undеrrеprеsеntеd in clinical trials tеsting ULTs drugs. In our study, womеn constitutеd 44.5% of thе еnrollеd participants in thе CKD-HUA population (n=89/200), rеflеcting a lowеr еnrolmеnt ratе than mеn. Our еnrolmеnt of 89 fеmalе participants’ rеprеsеnts a mеaningful advancеmеnt toward еquitablе inclusion. Our samplе sizе, though not so largе, is sufficiеnt to justify thе sеx-stratifiеd findings, particularly whеn viеwеd considеring thе legacy of disparity and mеthodological rigor appliеd in subgroup analysеs. Futurе trials should build on these insights to furthеr closе еnrolmеnt gaps and еnsurе translational rеlеvancе across divеrsе populations.

In our study, we observed an association between SUA and lipid profile both before and after treatment with ULTs. Increasing SUA levels have been shown to be highly correlated with any sort of dyslipidemia like increased LDL-c as well as decreased HDL-c. This corresponds with studies that indicate SUA has the capacity to increase oxidative stress and inflammation inhibiting lipid metabolism and enhancing atherogenic profiles (58). Furthermore, the anti-inflammatory effect of ULT may normalize HDL-c function and help increase reverse cholesterol transport. Notably, Male patients had a greater extent of improvements in lipids after ULTs which may be in part due to possible effects of hormones on SUA elimination and lipid metabolism.

While this investigation provides insight and evidence on the impact of ULTs on lipid profiles, certain limitations do need to be stated. First, the study is observational instead of a randomized controlled trial which could independently affect metabolic outcomes, and limit our ability to conclusively explore the effects of ULTs on sex and baseline characteristics and unmeasured variables. In our cohort study adjusting for demographics and laboratory parameters (eGFR, SUA, and lipid profiles), we acknowledge unmeasured confounders such as dietary purines, and genetic variants may still exist. Furthermore, we performed sensitivity analyses excluding patients with gout (which is a major modifier of SUA) and stratified analyses by metabolic syndrome status to assess robustness. Besides, the sample size (n=200), though adequate to detect moderate effect sizes, may lack the power to identify smaller yet clinically meaningful differences in lipid parameters or subgroup-specific effects. Furthermore, the study is from one city which limits the generalizability to broader populations, particularly those with demographic or clinical characteristics differing from our cohort. These limitations underscore the need for a cautious interpretation of associations and highlight the value of future prospective studies or randomized controlled trials that are necessary to validate these findings.

## 5 Conclusion

This observational cohort study demonstrated that ULT use was associated with improved lipid profiles, specifically lower LDL-c, TG and TC levels, and higher HDL-c levels in CKD stages 3/4 patients with hyperuricemia. Changes in the lipid profile appeared to be sex-dependent as there were greater reductions in LDL-c and increases in HDL-c in males than in females. Furthermore, ULT exposure also appeared to be associated with slower progression of CKD. Collectively, these findings highlight ULT’s potential role in managing dyslipidemia and renal decline in non-dialysis CKD and should be validated in randomized trials.

## Data Availability

The raw data supporting the conclusions of this article will be made available by the authors, without undue reservation.
