# Correction: Assessment of urate-lowering therapies on lipid metabolism and kidney function in nondialysis chronic kidney disease patients: 12 months multicenter cohort study

**DOI:** 10.3389/fendo.2026.1788912

**Published:** 2026-01-27

**Authors:** Yousuf Abdulkarim Waheed, Huanhuan Yin, Jie Liu, Shifaa Almayahe, Maryam Bishdary, Karthick Kumaran Munisamy Selvam, Syed Muhammad Farrukh, Shulin Li, Disheng Wang, Xinglei Zhou, Dong Sun

**Affiliations:** 1 Department of Nephrology, Affiliated Hospital of Xuzhou Medical University, Xuzhou, China; 2 Clinical Research Center for Kidney Disease Xuzhou Medical University, Xuzhou, China; 3 Department of Nephrology, Fengxian People’s Hospital, Xuzhou, China; 4 Department of Nephrology, the Second Affiliated Hospital of Xuzhou Medical University, Xuzhou, China; 5 Medical College, University of Fallujah, AL Anbar, Iraq; 6 Central Michigan University College of Medicine, Mount Pleasant, MI, United States; 7 Medical College, Xuzhou Medical University, Xuzhou, China; 8 Department of Internal Medicine and Diagnostics, Xuzhou Medical University, Xuzhou, China

**Keywords:** serum uric acid, chronic kidney disease, urate-lowering therapy, hyperuricemia, dyslipidemia, cardiovascular risk

Wrong affiliation, but not replacing with present address.

Affiliation ^4^ “Department of Nephrology, the Second Affiliated Hospital of Xuzhou Medical University, Xuzhou, China” was erroneously given as Affiliation ³ “Department of Nephrology, Fengxian People’s Hospital, Xuzhou, China” for author Xinglei Zhou.

The original version of this article has been updated.

